# Solution Combustion Synthesis of Cr_2_O_3_ Nanoparticles and the Catalytic Performance for Dehydrofluorination of 1,1,1,3,3-Pentafluoropropane to 1,3,3,3-Tetrafluoropropene

**DOI:** 10.3390/molecules24020361

**Published:** 2019-01-20

**Authors:** Haili Wang, Wenfeng Han, Xiliang Li, Bing Liu, Haodong Tang, Ying Li

**Affiliations:** Institute of Industrial Catalysis, Zhejiang University of Technology, Zhejiang 310032, China; wanghailii@outlook.com (H.W.); lxl199458@163.com (X.L.); 17326058928@163.com (B.L.); tanghd@zjut.edu.cn (H.T.); liying@zjut.edu.cn (Y.L.)

**Keywords:** solution combustion synthesis, Cr_2_O_3_, dehydrofluorination, HFC-245fa, HFO-1234ze, nanoparticles

## Abstract

Cr_2_O_3_ nanoparticles were prepared by solution combustion synthesis (SCS) with chromium nitrate as the precursor and glycine as the fuel. Commercial Cr_2_O_3_ and Cr_2_O_3_ prepared by a precipitation method were also included for comparison. The morphology, structure, acidity and particle size of fresh and spent Cr_2_O_3_ catalysts were investigated by techniques such as XRD, SEM, TEM, BET and NH_3_-TPD. In addition, catalytic performance was evaluated for the dehydrofluorination of 1,1,1,3,3-pentafluoropropane (CF_3_CH_2_CHF_2_, HFC-245fa) to 1,3,3,3-tetra-fluoropropene (CF_3_CH=CHF, HFO-1234ze). The catalytic reaction rate of Cr_2_O_3_ prepared by SCS method is as high as 6 mmol/h/g, which is about 1.5 times and 2 times higher than that of precipitated Cr_2_O_3_ and commercial Cr_2_O_3_, respectively. The selectivity to HFO-1234ze for all the catalysts maintains at about 80%. Compared with commercial and precipitated Cr_2_O_3_, Cr_2_O_3_-SCS prepared by SCS possesses higher specific surface area and acid amount. Furthermore, significant change in the crystal size of Cr_2_O_3_ prepared by SCS after reaction was not detected, indicating high resistance to sintering.

## 1. Introduction

1,1,1,3,3-Pentafluoropropane (CF_3_CH_2_CHF_2_, HFC-245fa) is a typical hydrofluorocarbon (HFC). It is mostly used as a physical foaming agent [1,2,3]. Due to its zero ozone depletion potential (ODP), HFC-245fa is being considered a third-generation foaming agent. However, its global warming potential (GWP) is about 1030 times higher than that of CO_2_. Therefore, HFC-245fa is controlled as a potent greenhouse gas by Kyoto Protocol and its various amendments. Recently, HFC-245fa was suggested to be the feedstock for producing 1,3,3,3-tetrafluoropropene (CF_3_CH=CHF, HFO-1234ze). It is an effective way for the sustainable development of HFCs. With ODP of 0 and GWP of only 6, HFO-1234ze is considered as one of the new generation of refrigerants and heat transfer working fluids [4]. In addition, HFO-1234ze is also applied in other fields, for example as a monomer for the synthesis of stable and elastic rubber plastics, a raw material for the preparation of agricultural chemicals, and a fire-proof protective gas for melting magnesium or magnesium alloy [5].

Actually, catalytic dehydrofluorination of HFC-245fa is one of the major routes to manufacture HFO-1234ze following the reaction indicated in Equation (1):

CF_3_CH_2_CHF_2_ (HFC-245fa) → CF_3_CH=CHF [(HFO-1234ze)] + HF (1)

In addition, HFO-1234ze can be converted to 2,3,3,3-tetrafluoropropylene (CF_3_CF=CH_2_, HFO-1234yf) in the presence of a suitable catalyst. HFO-1234yf is also a novel refrigerant [6,7,8]. However, as a major by-product of dehydrofluorination, corrosive HF poses significant challenges for the stability of catalysts. To survive in a highly corrosive HF atmosphere, metal fluorides, such as AlF_3_, metal oxides such as Cr_2_O_3_ and activated carbon (AC) were explored as the catalysts. Due to the abundant pores, activated carbon as the catalyst exhibits high activity. However, activated carbon is difficult to recover after deactivation due to the carbon deposition, which limits its commercial application.

It was found that the traditional catalysts adopted in fluorochemical industry, such as Cr_2_O_3_ and AlF_3_, present high activity for the conversion of HFC-245fa [9,10,11]. However, due to the strong acidity of these catalysts, coke deposition is a major challenge, leading to the rapid deactivation. In order to improve the performance of these catalysts, metal components such as Rh, Ni, Mg and Pd are adopted as the effective promoters to reduce coke deposition and increase the lifespan of the catalysts [10,11,12,13]. However, the addition of precious metals such as Rh and Pd significantly increases the cost of the catalysts. Introduction of oxygen into the feeding gas is one of the solutions to avoid the coke deposition and deactivation [8,14]. However, oxygen introduction leads to the loss of HFC-245fa and products, as HFC-245fa and products react with oxygen forming CO and CO_2_. In addition, the presence of oxygen also increases the cost of separation.

To inhibit the coke deposition and improve the performance of catalysts, the nanoscale or mesoporous catalysts were prepared by various methods [15,16,17]. At present, research is intensively focused on the preparation of nano-Cr_2_O_3_ [18,19]. With SBA-15 or Ca_3_(PO_4_)_2_ as the hard templates, Sun et al. [20] prepared Cr_2_O_3_ nanorods or nanoparticles with high specific surface areas. Mouni Roy et al. [21] synthesized Cr_2_O_3_ nanocubes with porous structure by solvothermal method. In our previous work [22], we fabricated nano-Cr_2_O_3_ catalyst successfully by solution combustion synthesis (SCS) method. The nano-Cr_2_O_3_ exhibits improved catalytic dehydrofluorination of 1,1-difluoroethane (HFC-152a, CH_3_CHF_2_) to the monomer of vinyl fluoride (VF, CH_2_=CHF). To ensure the complete formation of crystallized Cr_2_O_3_, the catalyst was calcined in air at 500 °C. Unfortunately, as reported, the calcination at 400 °C led to the increase in the particle size by more than 50% [23]. In addition, the pre-fluorination by CHClF_2_ before reaction also results in the partial coke deposition.

Precipitation is one of the most conventional routes for the preparation of oxide catalyst. However, precipitation usually produces significant amounts of waste solution. In addition, it is difficult to achieve uniform nanoparticles. By contrast, it is well accepted that the solution combustion synthesis is an important method for preparing nano-catalysts [24,25,26]. SCS method is simple, convenient and scalable. For the preparation of catalysts, SCS is mainly carried out by heating corresponding metal nitrates as oxidants and desired amounts of organics as the fuels through combustion. Following SCS, the product is usually crystallized with high surface area. During the combustion, large amounts of gases are produced which flush the solid agglomerates resulting in the fine powder. Herein, we synthesize Cr_2_O_3_ catalyst by SCS method with chromium nitrate as the precursor and glycine as the fuel for the dehydrofluorination of HFC-245fa to HFO-1234ze. It is emerging as one of the new generations of refrigerant and heat transfer working fluid. In the present investigation, Cr_2_O_3_ was evaluated as the catalyst for the catalytic dehydrofluorination of 1,1,1,3,3-pentafluoropropane (CF_3_CH_2_CHF_2_, HFC-245fa) to HFO-1234ze. It provides a potential way for the preparation value added HFO-1234ze. To avoid the sintering, no calcination of catalyst was adopted. Also, Cr_2_O_3_ tends to be partially fluorinated by the reactant, HFC-245fa and the dehydrofluorination product, HF. Consequently, different from the previous study, no pre-fluorination treatment was adopted.

## 2. Results and Discussion

### 2.1. Evaluation of Catalytic Activity

**Figure 1:**
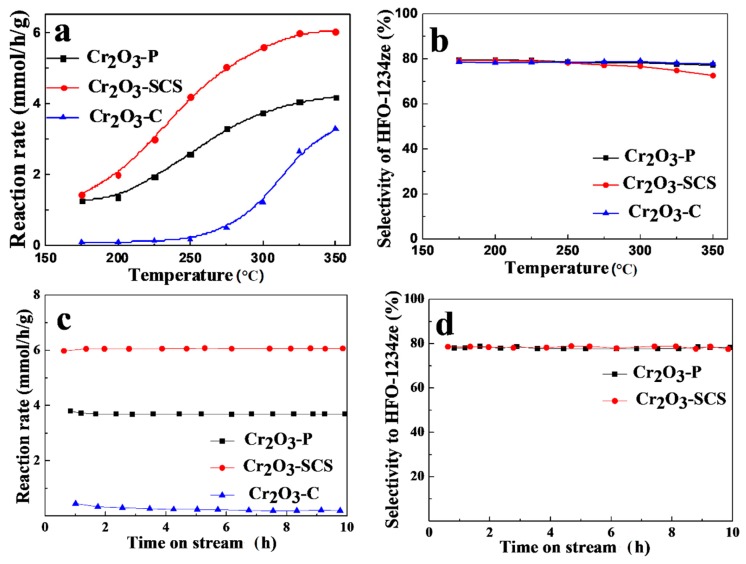
The performance of Cr_2_O_3_ catalysts obtained by precipitation method (P), solution combustion synthesis (SCS) and commercial catalyst (C) for the pyrolysis of HFC-245fa. (**a**) The conversion rate of HFC-245fa as a function of reaction temperature and (**b**) the selectivity to HFO-1234ze as a function of reaction temperature. (**c**) The conversion rate of HFC-245fa and (**d**) the selectivity to HFC-1234ze as a function of time on stream at 300 °C. Reaction conditions: 1 atm, N_2_: HFC-245fa of 1:4, GHSV (HFC-245fa) of 150 h^−1^.

The catalytic activities of Cr_2_O_3_ samples prepared by precipitation method (denoted as Cr_2_O_3_-P), solution combustion synthesis (SCS) and commercial Cr_2_O_3_ (denoted as Cr_2_O_3_-C) for the pyrolysis of HFC-245fa to HFO-1234ze are shown in Figure 1. Pyrolysis of HFC-245fa was carried out at the pressure of 1 atm and GHSV (gas hourly space velocity, HFC-245fa) of 150 h^−1^. During the reaction, HFO-1234ze was detected as the major product. Minor by-products include HFO-1234yf and trace amounts of 3,3,3-trifluorine-1-propyne (CF_3_CF≡CH_2_). As displayed in Figure 1a, the conversion level of HFC-245fa increases with reaction temperature significantly for all the catalysts. The Cr_2_O_3_-P and Cr_2_O_3_-SCS catalysts commence to catalyze the decomposition of HFC-245fa at temperatures below 175 °C. By contrast, the Cr_2_O_3_-C catalyst starts to promote the reaction at temperatures above 250 °C. Clearly, Cr_2_O_3_-SCS catalyst exhibits highest activity among these catalysts. Furthermore, the activity differs dramatically between Cr_2_O_3_-SCS and the other two catalysts with the increase in reaction temperature. The conversion rate of HFC-245fa over Cr_2_O_3_-SCS catalyst is about 1.5 mmol/h/g and close to 6 mmol/h/g at reaction temperature of 175 °C and 350 °C, respectively. Clearly, the reaction rate of Cr_2_O_3_-SCS catalyst is about 1.5 times and 2 times higher than that of precipitated Cr_2_O_3_ and commercial Cr_2_O_3_ at 350 °C. The selectivity to HFO-1234ze over all catalysts maintain at about 80% at temperatures between 175 and 350 °C. As presented in Figure 1c,d, all the catalysts show stable catalytic performance within a time on stream (TOS) of 10 h at 300 °C. During the reaction, significant amounts of HF were produced which may react with Cr_2_O_3_ changing the composition of the catalyst. Consequently, Cr_2_O_3_ was partially converted to CrOxFy by HF. It was suggested that CrOxFy is the active species in dehydrofluorination reactions [27,28,29]. Therefore, Cr_2_O_3_ exhibits stable activity in HF atmosphere.

### 2.2. Morphology and Structure of Cr_2_O_3_ Catalysts

**Figure 2:**
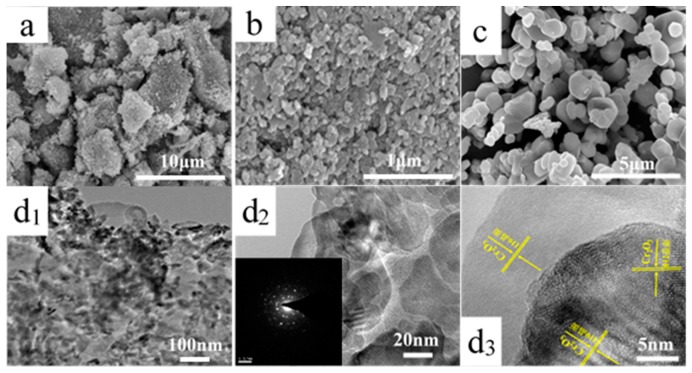
Morphology of Cr_2_O_3_ catalyst prepared by different methods. SEM images of (**a**) Cr_2_O_3_-P, (**b**) Cr_2_O_3_-SCS and (**c**) Cr_2_O_3_-C; (**d_1_**), (**d_2_**) and (**d_3_**) TEM images of Cr_2_O_3_-SCS with different magnification and the inset in (**d_2_**) shows the corresponding SAED patterns.

The morphology and structure of catalysts were investigated by scanning electron microscopy (SEM) and transmission electron microscopy (TEM), and the results are displayed in Figure 2. Due to the large amounts of gases released by glycine combustion, the surface of Cr_2_O_3_-SCS catalyst appears to be rather rough and porous. Abundant pores provide higher specific surface area which is a key parameter affecting the catalytic performance. Furthermore, the particle size is less than 100 nm, which is much smaller than the other two catalysts. The small particle size improves the exposure of catalyst surface, providing much more active sites. Thus, developed pores and small particles of Cr_2_O_3_-SCS catalyst result in higher catalytic activity. There are no clear porous channels on the surface of Cr_2_O_3_-P and larger particles are observed. The particle of Cr_2_O_3_-C is irregular, smooth and solid, which may partly contribute to the low catalytic performance over Cr_2_O_3_-C catalyst. 

To further investigate the micro-structure of Cr_2_O_3_-SCS catalyst, TEM characterization was carried out, and the images are demonstrated in Figure 2d_1_,d_2_ and d_3_. Clearly, Cr_2_O_3_-SCS is composed of ultra-fine particles (Figure 2d_1_). As displayed in Figure 2d_3_, the distances between the lattice fringes are confirmed to be 0.363, 0.245 and 0.167 nm, respectively, which are assigned to the d-spacing values of the (111) and (200) planes in Cr_2_O_3_ crystalline. According to the selected area electron diffraction (SAED) patterns in Figure 2d_2_, a large number of diffraction points are arranged around the center point, indicating that Cr_2_O_3_-SCS is close to the single crystal structure. In addition, there are also many disorderly multiple diffraction points, implying that the surface of Cr_2_O_3_-SCS is partly covered by carbon produced during combustion.

In conclusion, differences in surface morphology and structure of the three catalysts may lead to the differences in specific surface area, which further affects the exposure level of active site and catalytic activity.

**Figure 3:**
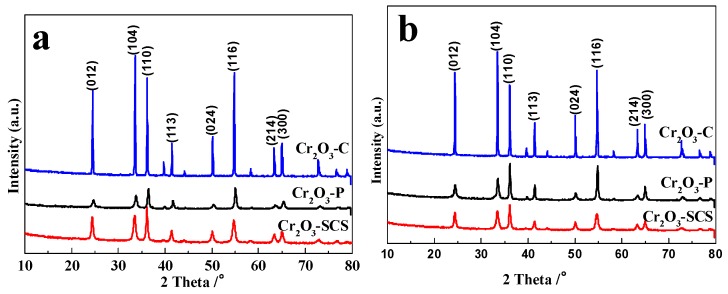
X-ray diffraction spectra for fresh Cr_2_O_3_ catalysts (**a**) and spent catalysts (**b**) prepared by different methods.

To elucidate the evolution of catalyst crystallization before and after the reaction, all catalysts were characterized by X-ray diffraction (XRD) and the results are demonstrated in Figure 3. As indicated in Figure 3, the XRD patterns of Cr_2_O_3_ derived from different preparation methods agree well with that of standard Cr_2_O_3_ pattern (PDF #38-1479, the space group: R-3c (167)), indicating that all samples possess the phase of Cr_2_O_3_ crystalline. In our previous study, the Cr_2_O_3_-SCS was calcined in air at 500 °C to obtain the complete crystal structure [22]. Clearly, calcination results in the partial sintering. Figure 3 conforms that that pure Cr_2_O_3_ can be derived without calcination. As a result, the particle size of Cr_2_O_3_-SCS is smaller than 100 nm, and while it is up to about 200 nm in our previous study. 

No other impurities were identified by XRD patterns. The sharp diffraction peaks imply that the as obtained samples are highly crystallized. However, the intensities of the crystal diffraction peaks based on (012), (104) (110), (113), (024), (116), (214) and (300) planes differ significantly among the three catalysts. Compared with Cr_2_O_3_-P and Cr_2_O_3_-SCS, although with identical diffraction peaks, the intensity of Cr_2_O_3_-C is much stronger, indicating Cr_2_O_3_-C catalyst possesses the highest degree of crystallinity among three catalysts. To further investigate the growth of Cr_2_O_3_ crystalline during reaction, we calculated the crystal sizes of fresh and spent Cr_2_O_3_ based on diffraction peaks of Cr_2_O_3_ phase. All the data of crystal sizes are calculated according to Scherrer formula [30] and the results are listed in Table 1.

The crystal size of Cr_2_O_3_-SCS catalyst increases slightly after reaction based on all the crystal facets. It indicates that the no significant sintering is observed for Cr_2_O_3_-SCS catalyst after time on stream of 10 h. By contrast, the crystal size of fresh Cr_2_O_3_-P catalyst is relatively larger than that of Cr_2_O_3_-SCS. In addition, it sinters significantly following reaction. Cr_2_O_3_-C has a crystal size of more than 100 nm and a larger particle size (It is out of the calculation range of Scherrer equation when the exact crystal size is larger than 100 nm).

In summary, the Cr_2_O_3_-SCS possesses higher sintering resistance, and while Cr_2_O_3_-P catalyst sinters facilely under reaction conditions. In addition, we suggest that the carbon produced in the process of combustion for Cr_2_O_3_-SCS catalyst prohibits the particle from growing.

**Figure 4:**
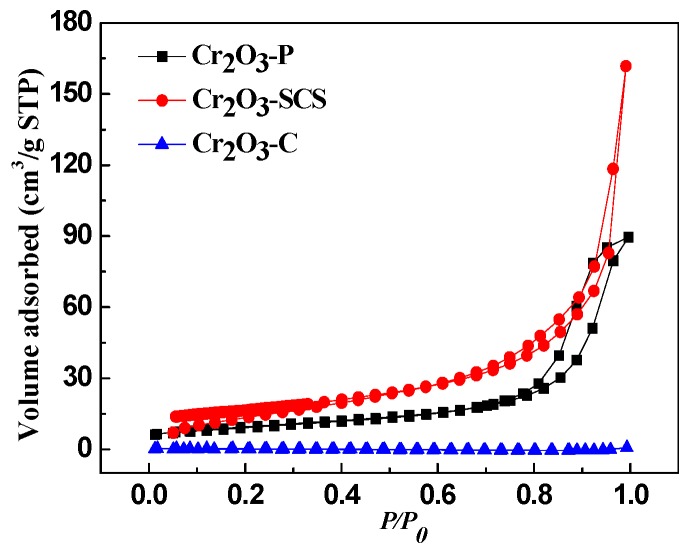
N_2_ adsorption isotherms of Cr_2_O_3_ catalysts prepared by different methods.

Figure 4 demonstrates the N_2_ adsorption-desorption isotherms for all the catalysts. The isotherms of Cr_2_O_3_-P and Cr_2_O_3_-SCS exhibit type IV characteristic (according to the IUPAC classification) with a well-defined capillary condensation step and H3 hysteresis loops which are usually observed with the aggregates of particles giving rise to slit-shape pores [31]. The pores are majorly generated between particles gaps. As expected, the Cr_2_O_3_-C catalyst shows a very low nitrogen adsorption, indicating the limited porosity. It is consistent with the SEM results that the surface of Cr_2_O_3_-C particles is very smooth. Furthermore, the Cr_2_O_3_-P and Cr_2_O_3_-SCS catalysts exhibit two capillary condensation steps. The capillary condensation step at relative pressure (*P*/*P*_0_) of 0.1–0.8 results from the adsorption of nitrogen in micropores, indicating that there are very few micropores in the pore walls between the adjacent nanorods. Another step at higher pressures (above 0.8) is derived from the adsorption of nitrogen in mesopores.

To confirm the effect of pore structure on catalytic performance, the textural parameters such as specific surface area, pore volume and pore size distribution are summarized in Table 2. As expected, the specific surface area of catalyst Cr_2_O_3_-C is as low as 0.6 m^2^/g, and while that of Cr_2_O_3_-SCS catalyst is as high as 58.2 m^2^/g. The specific surface area of Cr_2_O_3_-P is almost the average of them. Clearly, Cr_2_O_3_-SCS catalyst has developed pores compared with the other catalysts. It is consistent with SEM and TEM results that the Cr_2_O_3_-SCS catalyst with coarse surface possess a larger specific surface area, and a smaller specific surface area with smooth and non-porous surface over Cr_2_O_3_-C. As mentioned above, higher specific surface area usually provides more active sites. Thus, specific surface area and pore structure is one of the reasons for the difference in reaction rates over the three catalysts (Cr_2_O_3_-SCS > Cr_2_O_3_-P > Cr_2_O_3_-C).

It is worth noting that the surface area of Cr_2_O_3_-SCS is about 32 m^2^/g following calcination at 500 °C [22]. As displayed in Figure 3, the temperature during SCS is sufficient for the formation of Cr_2_O_3_ crystalline. Clearly, without calcination, sintering is avoided leading to improved surface area in this study.

### 2.3. Surface and Bulk Chemistry of Cr_2_O_3_ Prepared by Different Methods

**Figure 5:**
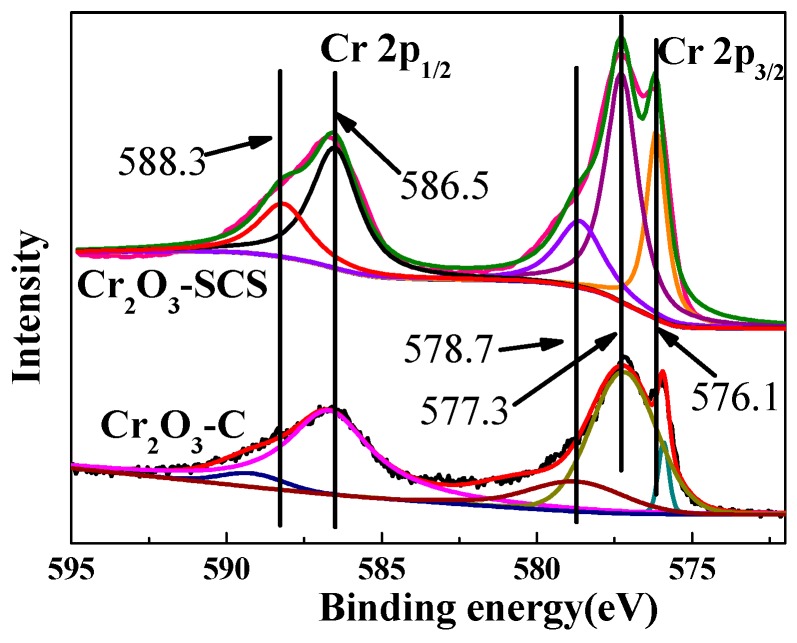
XPS characterization of Cr_2_O_3_-SCS and Cr_2_O_3_-C (Cr 2p_1/2_ and Cr 2p_3/2_).

Figure 5 presents the results of X-ray photoelectron spectroscopy (XPS) experiments for Cr_2_O_3_-SCS and Cr_2_O_3_-C. Very similar peaks are observed for both catalysts. According to the deconvolution of Cr 2p_3/2_ peaks, there are three Cr species both for Cr_2_O_3_-SCS and Cr_2_O_3_-C. The peak with binding energy of 576.1 eV indicates the existence of Cr(OH)_3_ [19,32] and the peak at binding energy of 577.3 eV is suggested to be the typical peak of Cr_2_O_3_ [33,34]. The peak at 578.7 eV is attributed to the CrO_3_ species [19,35]. Clearly, Cr(OH)_3_, Cr_2_O_3_, and CrO_3_ co-exist on the surface of Cr_2_O_3_-SCS and Cr_2_O_3_-C. The emergence of CrO_3_ is most probably attributed to oxidation of Cr_2_O_3_ at high temperatures.

Table 3 lists their respective surface compositions. According to the XPS results, the dominant phase on the surface of the sample is confirmed to be Cr_2_O_3_. The Cr_2_O_3_-SCS catalyst has more CrO_3_ phase. It may be resulted from the combustion of glycine which produces high temperature instantaneously facilitating the oxidation of Cr_2_O_3_. Another phase, Cr(OH)_3_ transforms into Cr_2_O_3_ readily at temperature above 300 °C. Hence, Cr(OH)_3_ plays a role in the formation of Cr_2_O_3_ although there is noticeable difference between Cr_2_O_3_-SCS and Cr_2_O_3_-C. However, the significant difference in CrO_3_ contents on the surface catalyst plays a major role in the catalytic performance as high-valent Cr species such as Cr (VI) are vital for the reaction because they could be transformed to the active species such as CrOxFy. As demonstrated in Table 4, the spent Cr_2_O_3_-SCS contains significant amounts of fluorine element, indicating that there are species such as CrOxFy in the surface.

**Figure 6:**
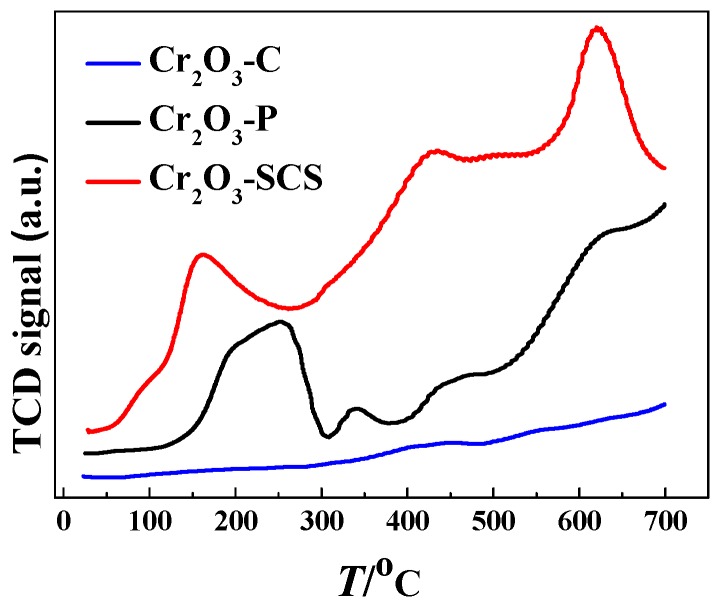
The profiles for temperature-programmed desorption of ammonia (NH_3_-TPD) for Cr_2_O_3_ catalysts prepared by different methods.

It is generally accepted that the active site of dehydrofluorination reaction is the surface acidic site of the catalyst [36]. Therefore, the activity of catalyst increases with surface acid content. Unfortunately, the acidic site is also the coke deposition center which is majorly responsible for the stability of catalyst [37,38,39]. The surface acidity of catalysts was characterized by temperature-programmed desorption of ammonia (NH_3_-TPD) as illustrated in Figure 6. The NH_3_-TPD is usually used for the investigation of the acid strength and acid amount on the surface of catalysts. A broad desorption profile in the range of 100–700 °C in Cr_2_O_3_-SCS is observed with three peaks at around 60 °C, 430 °C and 620 °C respectively. Five peaks appear in Cr_2_O_3_-P at around 195 °C, 255 °C, 340 °C, 450 °C, 620 °C. Desorption of NH_3_ on Cr_2_O_3_-C were minor.

Clearly, compared with Cr_2_O_3_-SCS and Cr_2_O_3_-P, Cr_2_O_3_-C contains very low level of acidity. As mentioned previously, acid sites are the active centers of dehydrofluorination reaction. Thus, it is reasonable that the reaction rate is very low over Cr_2_O_3_-C. Furthermore, the peak areas of NH_3_ desorption (represent the number of acidic sites) for the three catalysts were estimated. It is confirmed that the acid amount of Cr_2_O_3_-SCS is two times higher than that of Cr_2_O_3_-P, and about 8 times higher that of Cr_2_O_3_-C. It explains the results that Cr_2_O_3_-SCS has the highest catalytic activity, followed by Cr_2_O_3_-P. Therefore, acid content and acid species are the main reasons for the difference in catalytic activity of catalysts.

**Figure 7:**
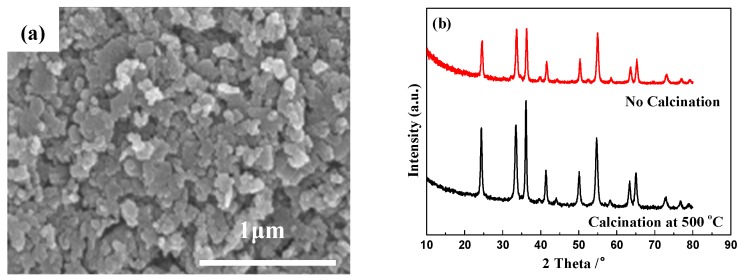
SEM image of Cr_2_O_3_-SCS following calcination at 500 °C for 2 h (**a**) and XRD patterns of Cr_2_O_3_-SCS before and after calcination at 500 °C for 2 h (**b**).

As discussed previously, different from our previous study [22], no calcination was adopted during the catalyst preparation. Clearly, as demonstrated in Figure 1 and Figure 3, high activity and well crystallized Cr_2_O_3_ are achieved without calcination. As demonstrated in Figure 7a, following calcination at 500 °C for 2 h, compared with the sample without calcination (Figure 2b), significant sintering is observed. In addition, the XRD patterns (Figure 7b) reinforce the conclusion. Following calcination, although exact the same diffraction peaks were detected, the peak intensities increase dramatically, indicating the growth of Cr_2_O_3_ crystalline.

## 3. Materials and Methods

### 3.1. Catalysts Preparation

#### 3.1.1. Solution Combustion Synthesis Method

Similar to our previous work [22], Cr(NO_3_)_3_·9H_2_O (>99.5%, Aladdin Company, Shanghai, China) was used as the Cr_2_O_3_ precursor. In a typical experiment, 20 g Cr(NO_3_)_3_·9H_2_O was first dissolved in 75 mL distilled water. Under vigorous stirring, 12.5 g glycine (>99%, Aladdin Company) as the fuel was added and mixed well. Then, the mixed solution was condensed at 70 °C in a furnace. As a result, the gel-like paste was obtained. Following condensing, the paste was loaded to a microwave oven (800 W, 2.45 GHz, 23 L, Midea Company, Foshan, Guangdong, China. Assisted by microwave, large amounts of smoke were released and combustion flame was observed in about less than 3 min. Following combustion, the sample was cooled to room temperature and green foam-like Cr_2_O_3_ powder was obtained. The sample is denoted as Cr_2_O_3_-SCS.

#### 3.1.2. Precipitation Method

As a comparison, Cr_2_O_3_ was also prepared by conventional precipitation method. 0.1 mol Cr(NO_3_)_3_·9H_2_O was first dissolved in 200 mL distilled water and an excessive amount of aqueous ammonia solution was added stepwise. After vigorous stirring for 2 h, the solution was filtered by Buchner funnel with a vacuum pump. Then the obtained solid was dried at 80 °C for 12 h. The sample is denoted as Cr_2_O_3_-P.

#### 3.1.3. Commercial Cr_2_O_3_

Analytically pure Cr_2_O_3_ received from Aladdin Company was adopted as a reference catalyst. The samples were ground and pressed into pellets at 20 MPa followed by crushing and sieving Cr_2_O_3_ particles between 0.4 and 0.8 mm. The sample is denoted as Cr_2_O_3_-C.

### 3.2. Catalytic Activity

Catalytic activity evaluation for dehydrofluorination reaction of 1,1,1,3,3-pentafluoropropane (HFC-245fa) to HFO-1234ze was carried out with a fixed-tube reactor (stainless steel with i.d. of 7.5 mm, Golden Eagle Technology, Tianjin, China. Cr_2_O_3_ catalyst (2 mL) was loaded into the isothermal zone of the reactor. A thermal couple in the middle of the catalyst bed functioned as the detector of the reaction temperature. Prior to reaction, the reactor was first purged with pure nitrogen to remove water vapor and air at reaction temperatures. The gas-phase HFC-245fa with GHSV (gas hourly space velocity) of 150 h^−1^, balanced by four times of nitrogen, passed through the reactor. Prior to the experiments, all the catalysts were ground and pressed into pellets at 20 MPa followed by crushing and sieving. Cr_2_O_3_ particles between 0.3 and 0.7 mm were collected and were loaded to the reactor. The gaseous effluent from the reactor passed through a scrubber containing about 1 M KOH solution (850 mL) to remove HF, followed by the composition analysis with a GC-9790 gas chromatograph (Fuli Instruments, Taizhou, Zhejiang, China) equipped with a thermal conductivity detector (TCD).

### 3.3. Catalyst Characterization

SEM images were used for the investigation of morphology were obtained on a FESEM system (Hitachi S-4700, Hitachi, Tokyo, Japan) with the accelerating voltage of 15 kV. It is equipped with an EDS. XRD patterns of catalysts were obtained by an X’Pert Pro analytical instrument (PANalytical B.V., Almelo, Netherlands). XPS was conducted at 3 mA and 15 kV on ESCALAB MkII (Waltham, MA, USA). To avoid the surface charging effect, binding energies were referenced to C1s binding energy of carbon, taken to be 284.6 eV. The XPS spectra were analyzed by the XPS peak software (XPS PEAk Fit 4.1, Systat Software, Inc., San Jose, CA, USA). TEM images included to further investigate the microstructure of catalysts were obtained with a 2100F Transmission Electron Microscope (JEOL, Akishima, Tokyo, Japan) at an acceleration voltage of 200 kV. The BET surface area and total pore volume of catalysts were measured by N_2_ adsorption-desorption at −196 °C with Quantachrome Autosorb Automated Gas Sorption System (Quantachrome, Boynton beach, FL, USA). The catalyst samples were degassed at 200 °C for 6 h under vacuum before measurements. NH_3_-TPD was carried out in a self-made instrument and a thermal conductivity detector (TCD) was used for detecting the NH_3_ signal.

## 4. Conclusions

The Cr_2_O_3_ catalyst was successfully prepared by solution combustion synthesis method with Cr(NO_3_)_3_·9H_2_O as the Cr precursor and glycine as the fuel. In addition, the catalysts was evaluated for the dehydrofluorination of HFC-245fa producing HFO-1234ze. Cr_2_O_3_ prepared by SCS exhibits very high reaction rate of HFC-245fa with 6 mmol/h/g, which is about 1.5 times and 2 times higher than that of precipitated Cr_2_O_3_ and commercial Cr_2_O_3_, respectively. It remains stable after 10 h time on stream. Compared with commercial and precipitated Cr_2_O_3_, Cr_2_O_3_ prepared by SCS possesses higher specific surface area and acid amount. Furthermore, no significant sintering of Cr_2_O_3_ prepared by SCS under reaction was detected, indicating high resistance to sintering.

## Figures and Tables

**Table 1:** Crystal size changes of fresh and spent Cr_2_O_3_ catalyst prepared by different methods.

Samples	Crystal Size (nm)							
	(012)	(104)	(110)	(113)	(024)	(116)	(214)	(300)
Cr_2_O_3_-SCS-fresh	20.8	15.8	24.4	20.5	18.1	15.1	18.2	20.7
Cr_2_O_3_-SCS-spent	20.7	15.8	26.4	23.0	19.9	16.1	21.4	25.2
Cr_2_O_3_-P-fresh	18.1	18.5	27.8	34.4	16.6	26.3	14.1	20.1
Cr_2_O_3_-P-spent	22.4	24.3	38.3	38.1	20.4	35.5	22.7	34.9
Cr_2_O_3_-C-fresh	>100							
Cr_2_O_3_-C-spent	>100							

**Table 2:** Textural parameters of Cr_2_O_3_ prepared by different methods.

Samples	Surface Area (m^2^/g)	Pore Volume (cm^3^/g)	Average Pore Diameter (nm)
Cr_2_O_3_-SCS	58.2	0.3	17.2
Cr_2_O_3_-P	33.5	0.1	16.6
Cr_2_O_3_-C	0.6	-	-

**Table 3:** Surface composition of chromium oxides based on the deconvolution of XPS peaks (Cr 2p_3/2_).

Catalysts	Chromium Oxides, mol%		
	Cr(OH)_3_	Cr_2_O_3_	CrO_3_
Cr_2_O_3_-SCS	26.2	52.1	21.7
Cr_2_O_3_-C	6.9	77.3	15.8

**Table 4:** Surface composition of fresh and spent Cr_2_O_3_-SCS determined by X-ray energy spectrometer (EDS).

Catalysts	Weight/%			
	C	O	F	Cr
Cr_2_O_3_-SCS-fresh	16	23.6	0	60.4
Cr_2_O_3_-SCS-spent	9.5	17.9	8.0	64.6
